# Advocating for in-center hemodialysis patients via anonymous survey

**DOI:** 10.1097/MD.0000000000030937

**Published:** 2022-10-14

**Authors:** Arun Rajasekaran, Anand Prakash, Spencer Hatch, Yan Lu, Gary R. Cutter, Abolfazl Zarjou

**Affiliations:** a Division of Nephrology, Department of Medicine, University of Alabama at Birmingham, AL, USA; b Division of Biostatistics, University of Alabama at Birmingham, AL, USA.

**Keywords:** anonymous, education, feedback, hemodialysis, satisfaction, survey

## Abstract

We conducted an anonymous survey in 9 of our university affiliated outpatient dialysis units to address the concern that many in-center hemodialysis patients may not feel comfortable sharing their experiences. Major goals of this study: Investigating level of patient satisfaction with their care; Evaluating the subjective perception of the level of understanding of patients regarding pertinent issues of their disease and its management; Identifying potential avenues for care improvement. Survey was conducted in English, paper-based, with answer choices to individual questions for patient satisfaction and education graded using a 5-point Likert scale. Regarding potential areas of improvement, patients were asked to choose as many areas as deemed appropriate. To ensure anonymity, the completed surveys were folded and dropped into a box. Overall, 253 out of 516 (49%) screened patients were eligible and completed the survey. Patients expressed favorable responses regarding satisfaction (mean rating > 4 in each of 14 questions) and education (mean rating > 4 in 8 questions, > 3.5 in 2 questions) regarding hemodialysis. About 62% of overall study participants identified at least one area where they felt additional information would result in improvement of care. Our results indicate that patients undergoing outpatient hemodialysis were overall satisfied and had a good perceptive understanding about their health. Based on the patients’ input, strategies focused on addressing pain and discomfort, privacy, providing information about palliative care/hospice, mental health resources, and the process of kidney transplantation may promote improvement in overall quality of care.

## 1. Introduction

Candid and constructive feedback is invaluable to any industry attempting to improve its defined metrics and benchmarks, and healthcare is no exception.^[1]^ Traditionally, targeting laboratory values and similar metrics have been the cornerstone of providing quality care to patients undergoing in-center hemodialysis. However, it is increasingly evident that integrating patients’ experience into other metrics adds vital information, with a substantial potential of improving outcomes and overall quality of life.^[2–6]^ Despite this recognition, the paramount challenge is to identify and implement relevant surveys with a high participation rate to capture germane and implementable information.

In the United States, there are currently over half a million individuals with end-stage kidney disease (ESKD) on maintenance dialysis, with about 90% undergoing hemodialysis at a designated outpatient center.^[7]^ As the only disease-specific federal entitlement program in the United States, providing maintenance dialysis is a substantial financial responsibility on Medicare.^[8,9]^ To account for quality health care for all Americans undergoing maintenance dialysis, the In-Center Hemodialysis Survey Consumer Assessment of Healthcare Providers and Systems survey (ICH CAHPS) was developed, tested, and endorsed by national quality forum in 2007.^[10–12]^ The ICH CAHPS is a longer version than 2 other frequently used CAHPS surveys.^[2]^ The ICH CAHPS survey scores are now utilized for the overall patient experience star ratings and are considered as a factor in calculating reimbursement of dialysis centers.^[2,13]^ Nevertheless, ICH CAHPS does not cover all aspects of patient care on dialysis, is quite lengthy to fill, and has a high non-respondent rate.^[2]^ In fact, during its development the response rate was recorded at 46%,^[10]^ a rate that declined to 33% using aggregate data from the 2016 fall and 2017 spring.^[14]^ Although the lengthy survey may be a contributing factor, it is reasonable to consider whether apprehension pertaining to non-confidentiality of the survey influences patient participation and providing genuine responses.

In-center hemodialysis patients represent a particularly vulnerable patient population who may be reluctant to share their experiences honestly, given a fear that it will negatively impact their care. To account for this potential limiting factor, and to build on previous surveys performed on this patient population, we conducted a confidential questionnaire-based quality initiative study. Our study had 3 major objectives: to understand the level of satisfaction of patients with varying aspects of outpatient hemodialysis; to evaluate the subjective perception of the level of education/understanding of patients regarding issues that pertain to their overall health and wellbeing; and requesting patients’ input to identify predefined potential avenues for improvement of care. While anonymous surveys have been utilized in various aspects of health care and its delivery,^[15–18]^ this study is the among the first to investigate the potential of a multifaceted questionnaire-based survey with the central aim of improving overall patient experiences among patients undergoing in-center hemodialysis.

## 2. Materials and methods

### 2.1. Study design

This was an anonymous questionnaire-based quality-initiative clinical survey and was conducted in 9 DaVita outpatient hemodialysis centers affiliated with the University of Alabama at Birmingham (UAB). This study was approved by the Institutional Review Board (IRB) of UAB and the DaVita Clinical Research Committee. The study was designated a Quality Assurance/Quality Initiative by both entities (UAB and DaVita), and the need for a formal written informed consent was waived. The conduct of the study was in accordance with the guidelines set forth by the Declarations of Helsinki. A predefined survey was created that included 3 main domains: Satisfaction—comprising 14 individual questions, each rated using a 5-point Likert scale (1 = Very poor; 2 = Poor; 3 = Neutral; 4 = Good; 5 = Excellent); (Table 1); Education/knowledge level—comprising 10 individual questions. Patients were specifically asked to rate their subjective knowledge about each question using the same 5-point Likert scale (Table 2); Patient engagement/input—to identify predefined potential avenues for improvement of care—comprising 7 individual questions. Patients were asked to choose as many questions as deemed appropriate (minimum = 0, maximum = 7) by placing a check () or “X” () next to each individual question (Table 3). UAB nephrology physicians handed out the paper-based survey to patients after they were initiated on hemodialysis. Patients received verbal information about the rationale, anonymity, content of survey, and guidance on application of the scale to the first 2 sections (satisfaction, education/knowledge) and appropriate designation of answers to the last section (engagement/input). To enhance anonymity, the survey was conducted on paper and dropped into a box once completed. Among interested participants, only those capable of independently filling out the questionnaire on their own were surveyed. Those legally blind, unable to read/write/speak English, patients having advanced dementia, or too frail based on physician’s judgement were excluded from participation. The survey was conducted at all schedules at each dialysis center. This study was approved by the IRB of the UAB (UAB IRB approval number IRB-300008824). The conduct of the study was in accordance with the guidelines set forth by the Declarations of Helsinki.

**Table 1:** Patient satisfaction.

How satisfied are you with the overall care you receive regarding your dialysis?
How satisfied are you with dialysis improving your quality of life?
How satisfied are you with commute to and from your dialysis unit?
How satisfied are you with cleanness of your dialysis unit?
How often do dialysis center staff (nurses, technicians, dietitians, and social workers) listen carefully to you?
How often do dialysis center staff (nurses, technicians, dietitians, and social workers) explain things to you in a way that you fully understand?
How often do dialysis center staff (nurses, technicians, dietitians, and social workers) spend enough time with you to answer your questions?
How often do you feel your pain or discomfort is fully addressed during dialysis?
Are you satisfied with the amount of time that your doctor spends with you?
How often does your doctor explain things to you in a way that you fully understand?
How often does your doctor listen carefully to you?
How satisfied are you with your privacy at your dialysis center?
How satisfied are you with re-scheduling your dialysis if you miss a session?
How satisfied are you with your dialysis center staff addressing your mental health?

All questions rated using a 5-point Likert scale (1 = Very poor; 2 = Poor; 3 = Neutral; 4 = Good; 5 = Excellent).

**Table 2:** Patients’ subjective perception of education and knowledge.

Do you think you have enough knowledge about whether you are getting enough dialysis?
Do you think you have enough knowledge about why high phosphorus is bad for your health?
Do you know why high potassium in your diet can be dangerous for you?
Do you know what medicines are being given to you during dialysis?
Do you feel the results of your blood tests are explained to you in a way that you fully understand?
Do you feel you have received enough education about process of kidney transplantation?
Do you feel you have received enough education about doing dialysis at home?
Do you feel you have received enough education about palliative care or hospice?
Did you know that heart and blood vessel disease frequently affect dialysis patients?
Do you feel you have received enough education about what you should eat or drink?

All questions rated using a 5-point Likert scale (1 = Very poor; 2 = Poor; 3 = Neutral; 4 = Good; 5 = Excellent).

**Table 3:** Potential avenues for improvement.

More information about your diet
More information about why the labs discussed with your doctor are important
More information about your dialysis access
More information about transplant process
More information about mental health resources
Spend more time with your doctor
Spend more time with social worker and supporting staff

Patients may choose to answer as many questions as interested (minimum = 0, Maximum = 7) by placing a check () or “X” () next to each individual question.

### 2.2. Supplemental data analysis

We evaluated the survey results in a number of ways including simple distributions and counts univariately question by question. As with any user survey, there are individuals who are generally positive on all questions and a few who are thoroughly negative. It is of course difficult to assess whether individuals who are positive or negative to nearly all the questions are just either content or malcontent, or are simply trying to quickly finish a questionnaire. To assess how much consistent answering across the board was present in the survey, we created binary variables for each question on the 5-point Likert scale: marking a score of 4 (Good) or 5 (Excellent) to be coded as 1 (highly positive), and a score of anything less than 4 (1: Very poor, 2: Poor, or 3: Neutral) to be coded as 0 (not positive). We then compared the sum of scores overall, and within the 14 satisfaction questions and within the 10 education questions, individually. In addition, we looked for what might be considered behaviorally more truthful responses by comparing pairs of questions looking at discordant responses within the individual satisfaction and education sections. We also felt that the identification of the informative questions would come from the discordant responses between certain questions. We compared and tested these discordant pairs, which we felt might be identifying areas that may need improvement and are more indicative of potential factors on which to intervene, using McNemar’s test. *P* values <.05 were considered statistically significant. No adjustments were made for multiple comparisons, as we were looking for consistency rather than simply statistical significance. Further details are available in the supplemental material, http://links.lww.com/MD/H506.

## 3. Results

### 3.1. Details of study design, eligibility, and participation rate

A total of 516 patients undergoing outpatient hemodialysis were screened at 9 individual UAB affiliated dialysis units. Amongst them, 263 were excluded from the study. Among the excluded patients, 228 patients did not wish to participate, and 35 patients were excluded by the nephrology physician as these patients had one or more of the following exclusion criteria - legally blind, unable to read/write/speak English, had advanced dementia or were deemed too frail. A total of 253 patients (49%) were eligible and completed the survey.

### 3.2. Exploring the level of satisfaction of in-center hemodialysis patients with their care

**Figure 1.**
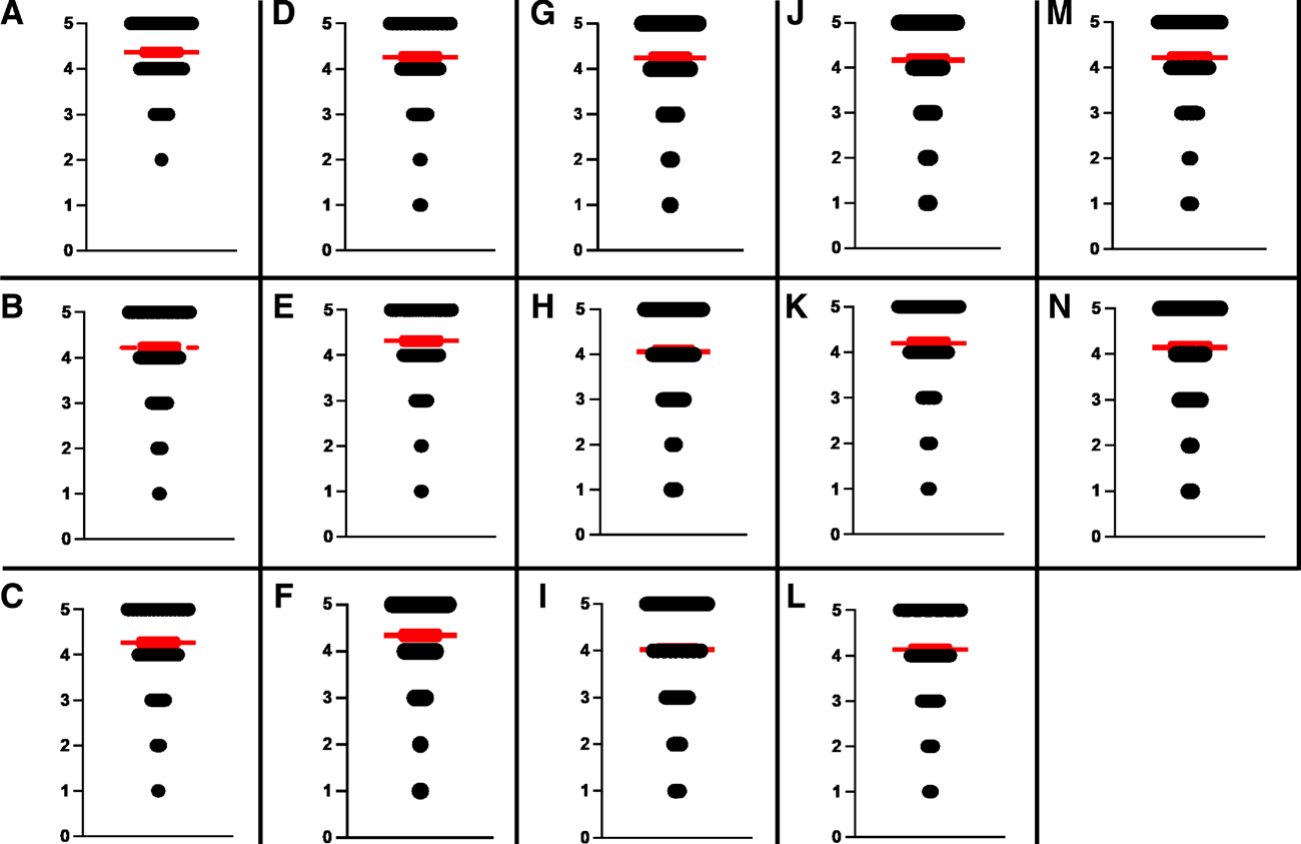
**In-center hemodialysis patients’ satisfaction with impact of their disease and management.** Panels A-N represent the following “Satisfaction” based questions. A: How satisfied are you with the overall care you receive regarding your dialysis? B: How satisfied are you with dialysis improving your quality of life? C: How satisfied are you with commute to and from your dialysis unit? D: How satisfied are you with cleanness of your dialysis unit? E: How often do dialysis center staff (nurses, technicians, dietitians, and social workers) listen carefully to you? F: How often do dialysis center staff (nurses, technicians, dietitians, and social workers) explain things to you in a way that you fully understand? G: How often do dialysis center staff (nurses, technicians, dietitians, and social workers) spend enough time with you to answer your questions? H: How often do you feel your pain or discomfort is fully addressed during dialysis? I: Are you satisfied with the amount of time that your doctor spends with you? J: How often does your doctor explain things to you in a way that you fully understand? K: How often does your doctor listen carefully to you? L: How satisfied are you with your privacy at your dialysis center? M: How satisfied are you with re-scheduling your dialysis if you miss a session? N: How satisfied are you with your dialysis center staff addressing your mental health? All questions rated using a 5-point Likert scale (1 = Very poor; 2 = Poor; 3 = Neutral; 4 = Good; 5 = Excellent) Width of black bar on individual score component represents corresponding number of respondents who opted for that particular score. Red bar represents mean individual score ± SEM. SEM = standard error of the mean.

Overall, patients expressed favorable responses regarding satisfaction, with a mean score > 4 in each of 14 questions (Fig. 1). High scoring areas in this category includes overall care received during dialysis (mean score 4.37, SEM ± 0.04), ability to understand things explained by the dialysis center staff (mean score 4.35, SEM ± 0.05), and satisfaction with the frequency of dialysis staff listening carefully to patients (mean score 4.37, SEM ± 0.05). Lower scoring areas in this section includes satisfaction with the amount of time spent by the physician (mean score 4.02, SEM ± 0.07), addressing pain or discomfort during dialysis (mean score 4.07, SEM ± 0.06), and satisfaction with privacy at the dialysis center (mean score 4.13, SEM ± 0.06).

### 3.3. Investigating degree of subjective perception of knowledge pertaining to ESKD, its complications, and components of care

**Figure 2.**
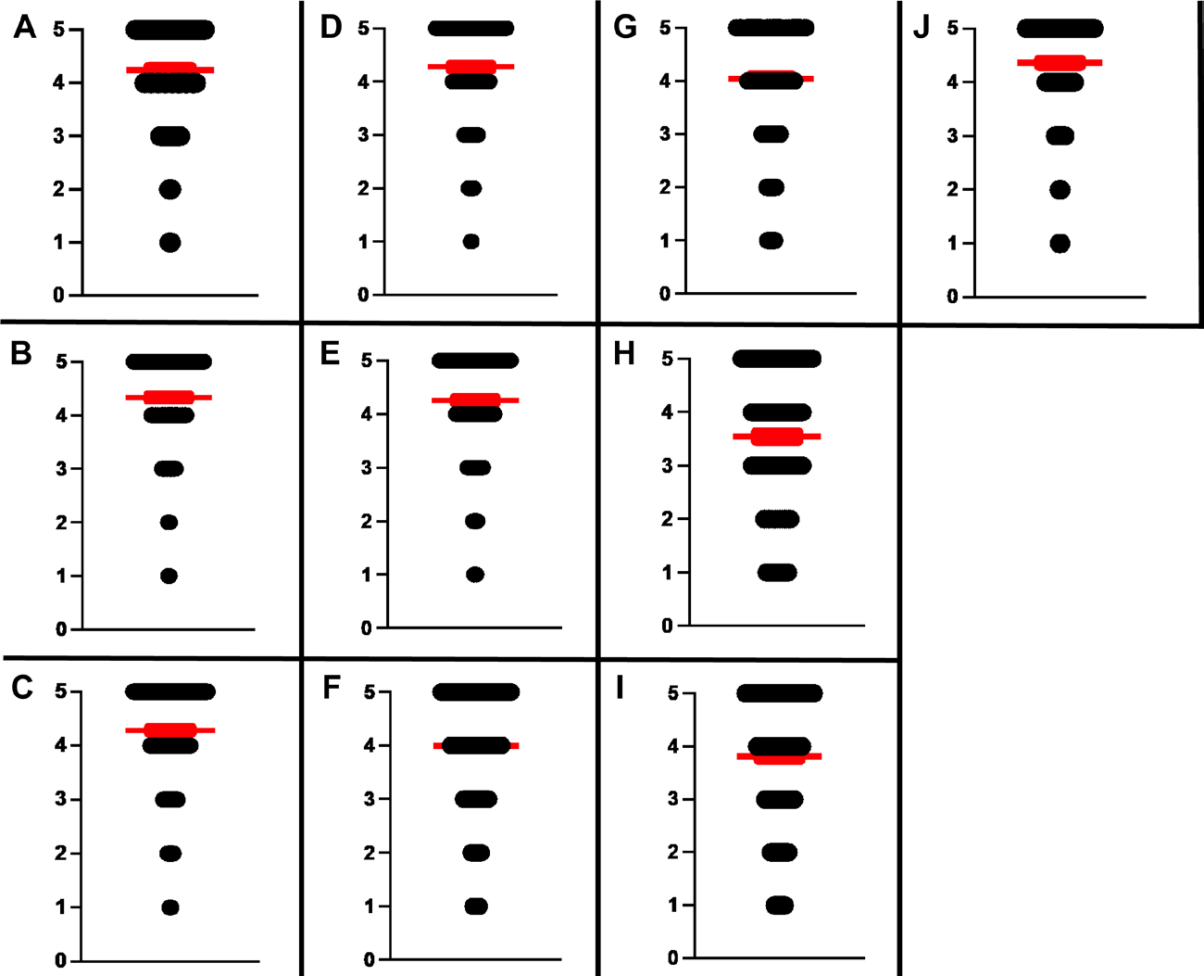
**In-center hemodialysis patients’ subjective perception of education and knowledge about various aspects of their disease and care.** Panels A-J represent the following “Education” based questions. A: Do you think you have enough knowledge about whether you are getting enough dialysis? B: Do you think you have enough knowledge about why high phosphorus is bad for your health? C: Do you know why high potassium in your diet can be dangerous for you? D: Do you know what medicines are being given to you during dialysis? E: Do you feel the results of your blood tests are explained to you in a way that you fully understand? F: Do you feel you have received enough education about process of kidney transplantation? G: Do you feel you have received enough education about doing dialysis at home? H: Do you feel you have received enough education about palliative care or hospice? I: Did you know that heart and blood vessel disease frequently affect dialysis patients? J: Do you feel you have received enough education about what you should eat or drink? All questions rated using a 5-point Likert scale (1 = Very poor; 2 = Poor; 3 = Neutral; 4 = Good; 5 = Excellent) Width of black bar on individual score component represents corresponding number of respondents who opted for that particular score. Red bar represents mean individual score ± SEM. SEM = standard error of the mean.

This section also revealed overall favorable responses with a mean score > 4 in 8 questions, and > 3.5 in 2 questions (Fig. 2). High scoring areas include education about appropriate diet and fluid intake (mean score 4.36, SEM ± 0.06), knowledge regarding the deleterious effects of elevated total body phosphorus (mean score 4.33, SEM ± 0.05), knowledge regarding the deleterious effects of high dietary potassium consumption (mean score 4.28, SEM ± 0.06), and knowledge regarding medications administered during dialysis (mean score 4.28, SEM ± 0.06). Low scoring areas include knowledge about palliative care or hospice (mean score 3.54, SEM ± 0.08), understanding about increased prevalence of cardiovascular disease amongst ESKD patients (mean score 3.82, SEM ± 0.07), and receiving enough education regarding process of kidney transplantation (mean score 4.00, SEM ± 0.07).

### 3.4. Engaging patients to identify predefined potential avenues for improvement of care

**Figure 3.**
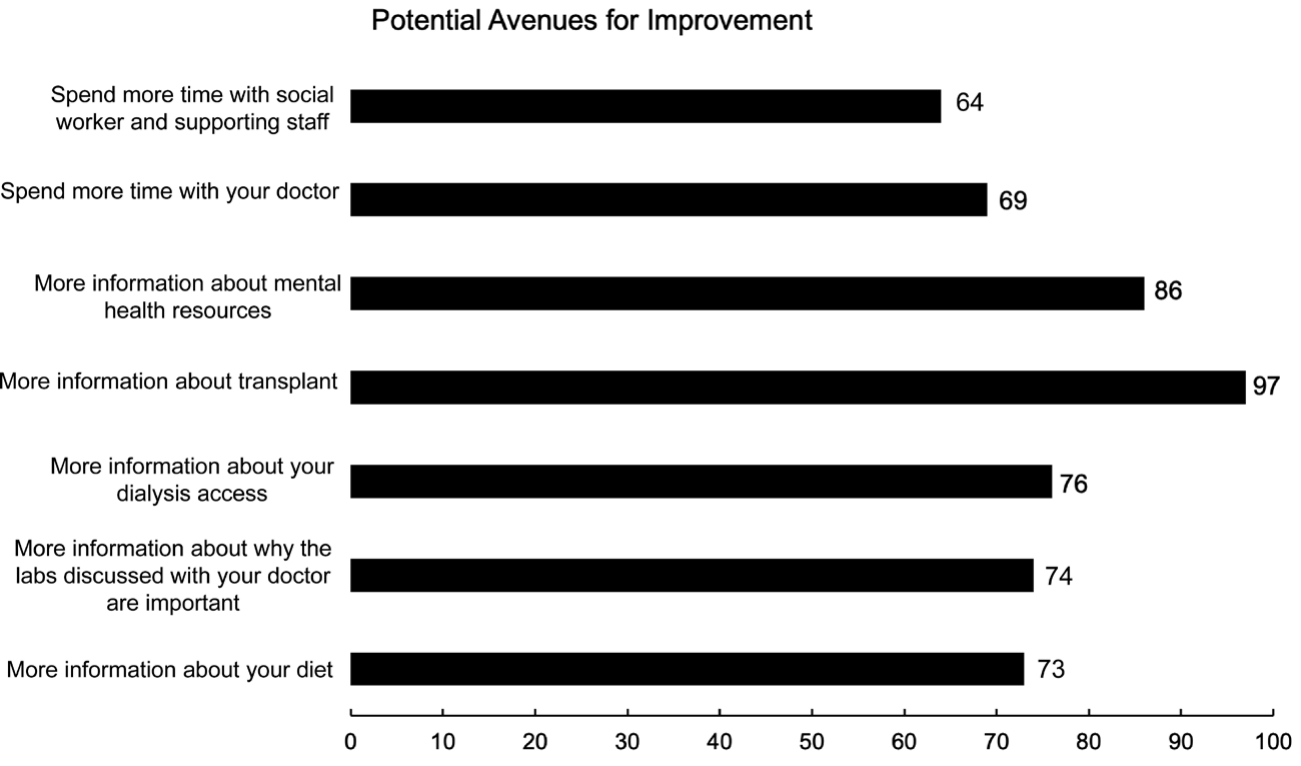
**Hemodialysis Patients’ input to identify predefined potential avenues for improvement of care.** Patients were allowed to choose and answer as many questions as they deemed appropriate (minimum = 0, Maximum = 7) by placing a check () or “X” () next to each individual question.

Data concerning this section of the survey are presented in Figure 3. Overall, 157 participants (~62% study participants) identified at least one area where they felt additional information would result in improvement of care. We demonstrate that 97 participants (~38%) suggested information regarding transplantation as a priority and this was closely followed by the request for more information about mental health resources (86 participants, ~34%). Interestingly, only 69 participants (~27%) identified spending more time with the attending nephrologist would translate to improvement in care.

### 3.5. Proportion of high (Scores of 4 or 5) versus low scoring (Scores < 4) individuals pertaining to satisfaction and education component of survey

Table 4 shows the proportion of responses that were scored high (4 or 5) by question with regards to the satisfaction component. Table 5 shows the proportion of responses that were scored high (4 or 5) by question with regards to the education component. Figure S1, http://links.lww.com/MD/H507 demonstrates the frequency distribution of the number of questions that were scored high for satisfaction and education component of the survey combined. Figure S2, http://links.lww.com/MD/H508 shows the frequency distribution of the number of questions that were scored high for satisfaction scores alone. Figure S3, http://links.lww.com/MD/H509 shows the frequency distribution of the number of questions that were scored high for education scores alone. Further details regarding individual scores where responses differed amongst the satisfaction questions and education questions are available in the supplemental material, http://links.lww.com/MD/H506.

**Table 4:** Proportion of high scoring pertaining to satisfaction.

Satisfaction questions with score 4 or 5	Proportion scoring 4 or 5	Standard deviation
How satisfied are you with the overall care you receive regarding your dialysis?	0.79	0.41
How satisfied are you with dialysis improving your quality of life?	0.81	0.40
How satisfied are you with commute to and from your dialysis unit?	0.82	0.39
How satisfied are you with cleanness of your dialysis unit?	0.80	0.40
How often do dialysis center staff (nurses, technicians, dietitians, and social workers) listen carefully to you?	0.85	0.36
How often do dialysis center staff (nurses, technicians, dietitians, and social workers) explain things to you in a way that you fully understand?	0.85	0.36
How often do dialysis center staff (nurses, technicians, dietitians, and social workers) spend enough time with you to answer your questions?	0.82	0.38
How often do you feel your pain or discomfort is fully addressed during dialysis?	0.74	0.44
Are you satisfied with the amount of time that your doctor spends with you?	0.72	0.45
How often does your doctor explain things to you in a way that you fully understand?	0.77	0.42
How often does your doctor listen carefully to you?	0.79	0.41
How satisfied are you with your privacy at your dialysis center?	0.76	0.43
How satisfied are you with re-scheduling your dialysis if you miss a session?	0.79	0.41
How satisfied are you with your dialysis center staff addressing your mental health?	0.73	0.44

High satisfaction score entails a score of either 4 (Good) or 5 (Excellent) on a 5-point Likert scale.

**Table 5:** Proportion of high scoring pertaining to education and knowledge.

Education questions with score 4 or 5	Proportion scoring 4 or 5	Standard deviation
Do you think you have enough knowledge about whether you are getting enough dialysis?	0.81	0.39
Do you think you have enough knowledge about why high phosphorus is bad for your health?	0.83	0.37
Do you know why high potassium in your diet can be dangerous for you?	0.82	0.39
Do you know what medicines are being given to you during dialysis?	0.79	0.41
Do you feel the results of your blood tests are explained to you in a way that you fully understand?	0.81	0.39
Do you feel you have received enough education about process of kidney transplantation?	0.72	0.45
Do you feel you have received enough education about doing dialysis at home?	0.75	0.43
Do you feel you have received enough education about palliative care or hospice?	0.54	0.50
Did you know that heart and blood vessel disease frequently affect dialysis patients?	0.65	0.48
Do you feel you have received enough education about what you should eat or drink?	0.79	0.41

High education score entails a score of either 4 (Good) or 5 (Excellent) on a 5-point Likert scale.

## 4. Discussion

In this study, we built on and created a modified version of the ICH CAHPS survey,^[10–12]^ and utilized a novel approach of implementing an anonymous survey in 9 of our university-affiliated outpatient hemodialysis centers. We endeavored to gain insight into potential areas that may merit additional resources and attention with the overall objectives of improving patient satisfaction, education, and quality of life. The participation rate among eligible individuals was 53%, and we were able to identify multiple aspects of treatment plans that could be tailored to better match patients’ preferences and needs.

Clinical and laboratory benchmarks pertaining to overall care of ESKD patients can be easily obtained and analyzed from the dialysis electronic medical records, with the results used to develop strategies aimed at improving patient care outcomes. In contrast, accurate assessment of the patient experience is more challenging, as it stems from multiple factors, including expectations, cultural differences, socioeconomic status, and health literacy. Mounting evidence corroborates that this objective can be achieved by including patient reported outcomes in the routine care of chronically ill patients.^[19–21]^ Ideally, the ensuing outcome should enhance engagement of patients in their overall care. The Beryl Institute defines patient experience as “the sum of all interactions, shaped by an organization’s culture, that influence patient perceptions across the continuum of care.”^[22]^ In non-dialysis health care settings, focusing on patient experience has been associated with improvements in clinical and overall financial outcomes.^[3,23,24]^ We reasoned that the challenging, and complex nature of ESKD and its management, may render a substantial number of such patients not to be entirely forthcoming about their struggles and various barriers they face. This reasoning led us to implement a confidential survey assessing 3 main domains.

First, we were interested to gauge the level of satisfaction of our ESKD patients with delivery of in-center hemodialysis. The highest scores were reported regarding the overall care, staff explaining issues in an understandable manner to patients, and staff listening carefully to the patients. In contrast, the lowest scores in this domain pertained to the amount of time physicians spent with patients, pain or discomfort being fully addressed during dialysis, and privacy during dialysis. Patients on hemodialysis are seen by their nephrologists and or advanced practice practitioners 1 to 4 times per month, although the contact time can vary widely. It is reported that the amount of time per visit by nephrology practitioners was very strongly associated with improved health outcomes, rather than the frequency of visits.^[25]^ In the outpatient hemodialysis setting, higher nephrology provider visit frequency was associated with fewer hospital admissions and earlier placement of permanent vascular access.^[26,27]^ However, in an analysis at the dialysis facility level with regards to patient experience, frequent nephrology practitioner visits to outpatient hemodialysis facilities were not associated with better patient experiences.^[28]^ This observation may be related to the fact that during high-visit frequency less time may be spent with each individual patient at the expense of spending ample time with those who may benefit the most from closer attention. Uncontrolled pain in patients receiving hemodialysis leads to shortened or missed treatments.^[29]^ ESKD associated pain is multifaceted and demands a comprehensive plan that implements pharmacological and non-pharmacological interventions. There is not much available in the literature on the importance and impact of patient privacy during dialysis, and based on our survey, additional studies in this area are warranted.

Second, we attempted to discern the degree of education and perceptive knowledge of patients concerning various aspects of their disease and care. We found that majority of our patients reported a good understanding about their ESKD dietary restrictions, detrimental effects of excessive phosphorus and medications routinely administered during dialysis. The lowest scores were related to knowledge about palliative care/hospice, ESKD associated cardiovascular diseases, and information pertaining to kidney transplantation. Rates of hospitalizations, Intensive Care Unit admissions, and death are substantially higher in older dialysis patients compared to the rest of older Medicare beneficiaries.^[30,31]^ Data suggests that fewer than 20% of U.S. dialysis patients are referred to hospice before death, compared with 55% of overall Medicare beneficiaries with cancer and 38.1% of those with heart failure.^[32]^ In addition, the median length of hospice is 5 days for those on dialysis, lower than the 17.4 days for the general Medicare population.^[33,34]^ A patient-centered approach will likely remove the stigma associated with hospice utilization and improve participation of ESKD patients when appropriate. Furthermore, it is increasingly clear that involvement of palliative care could serve as a complementary approach to providing quality care and should not merely be viewed as a mutually exclusive option.^[35,36]^ A recent study surveyed 172 family members’ understanding of the end-of-life wishes of patients undergoing maintenance hemodialysis, and showed that majority of family members reported having discussed about end-of-life preferences but not necessarily regarding discontinuing dialysis or hospice services. Despite the family members having a fair understanding of patients’ cardiopulmonary resuscitation (code status) wishes, a significant number lacked a complete understanding of their opinions on other domains pertaining to end-of-life care.^[37]^ Cardiovascular disease is the major etiology for morbidity and mortality in patients with ESKD.^[38,39]^ Cardiovascular disease is present in greater than 50% of patients undergoing hemodialysis, and the prevalence of coronary artery disease and left ventricular hypertrophy are around 40% and 70%, respectively.^[40]^ Involving patient advocates, handouts, recurrent emphasis in the form of annual or bi-annual classes to engage patients and their family members on prevalence and counseling on modifiable cardiovascular risk factors, and prompt referral to cardiology when indicated may prove beneficial. Many aspects of quality of life as well as cardiovascular survival drastically improves after kidney transplantation even in high-risk individuals.^[41–43]^ Therefore, it is not surprising that our surveyed patients felt they need more information and education about the gold standard care of ESKD treatment namely, kidney transplantation. Standardizing kidney transplant education may also alleviate some of the racial and socioeconomic disparities observed in kidney transplantation.^[44]^

Lastly, when encouraged to voice their preference, most respondents requested more information about kidney transplantation and mental health resources. Depression is the most common psychiatric illness in ESKD patients, with reported prevalence being 23% to 39%.^[43]^ Depression in ESKD patients remains under-recognized and under-treated.^[45]^ It is paramount that the dialysis team identify high-risk patients and promptly refer them to mental health providers for formal psychiatric assessment. Nephrologists need to work closely with their psychiatry counterparts to provide frequent counseling and patient-directed treatmen.^[46]^ At our institution, we recently commenced a dedicated psychology clinic for ESKD patients to address this specific issue.

Overall, we believe that this study provides critical information that can complement the ICH CAHPS’ survey for improving satisfaction, education, and engagement of our patient population. Strengths of this study include reasonably large sample size and participation rate, collection of data from multiple (9) dialysis centers, and the anonymous nature of the survey allowing patients to feel safe to participate. We also acknowledge that this study is rather an informal assessment which lacks standardization and hence precludes ability to compare the overall patient experience between patients, across providers, or possible trends over time. Furthermore, the anonymous nature of this survey precluded gathering any relevant information relating to patient demographics (including age, gender, race), socioeconomic status, time on hemodialysis, prior kidney transplant status, etc. Additionally, to ensure anonymity we obtained information only from English speaking patients who may not represent the non-English speaking population’s distinct needs. In the interest of simplicity, to allow maximal participation of all patients from all backgrounds and literacy levels, and to avoid input and interactions while filling the survey for the sake of anonymity, we implemented a 5-point Likert scale questionnaire with a simplistic design suitable and easily interpretable by the intended study population. We acknowledge that this approach may not be ideal for all the posed questions but accomplishes the objectives of this study.

It is also noteworthy that despite substantial progress in participation rate of eligible individuals in this study, close to 47% of eligible patients did not wish to participate in our survey. Engagement of more individuals to voice their opinion and provide candid and productive feedback remains a major challenge and priority. Based on our observations we suggest that development of tangible improvements and communications that are directly linked to patients’ input and responses may further enrich direct engagement of our patients in their care and delivering feedback. Therefore, addressing the identified areas of concern in this survey aligned with a follow up annual survey may provide a distinct platform to construct enhanced involvement of our patient population.

In summary, we reason those patients who receive in-center hemodialysis are particularly vulnerable and an anonymous approach to gauge their experience and knowledge level may be a useful tool to better understand the impact of illness and treatment. Such information enables designing enhanced strategies to advance the overall quality of life in this patient population.

## Acknowledgments

We wish to thank the clinical faculty and fellows of the Nephrology division at the University of Alabama at Birmingham. We also thank Dr Michael Allon for his critical review of this manuscript. We express our sincerest gratitude to the outpatient dialysis center directors for allowing us to conduct the survey in their units.

## Author contributions

Substantial contributions to conception and design, or acquisition of data, or analysis and interpretation of data: AR, AP, SH, YL, GC, AZ. Drafting the article or revising it critically for important intellectual content: AR, GC, AZ. Final approval of the version to be published: AR, AP, SH, YL, GC, AZ. Agreement to be accountable for all aspects of the work pre- and post-publication: AR, AP,SH, YL, GC, AZ.

**Conceptualization:** Arun Rajasekaran, Gary R. Cutter, Abolfazl Zarjou.

**Data curation:** Arun Rajasekaran, Anand Prakash, Spencer Hatch, Gary R. Cutter.

Formal analysis: Gary R. Cutter.

**Investigation:** Arun Rajasekaran, Anand Prakash, Spencer Hatch, Yan Lu, Gary R. Cutter, Abolfazl Zarjou.

**Methodology:** Arun Rajasekaran, Anand Prakash, Spencer Hatch, Yan Lu, Gary R. Cutter, Abolfazl Zarjou.

**Project administration:** Abolfazl Zarjou.

**Resources:** Abolfazl Zarjou.

**Software:** Gary R. Cutter.

**Supervision:** Gary R. Cutter, Abolfazl Zarjou.

**Validation:** Arun Rajasekaran, Gary R. Cutter, Abolfazl Zarjou.

**Visualization:** Arun Rajasekaran, Abolfazl Zarjou.

**Writing – original draft:** Arun Rajasekaran, Gary R. Cutter.

**Writing – review & editing:** Arun Rajasekaran, Gary R. Cutter, Abolfazl Zarjou.

## Supplementary Material


