# Supplementary material for: Advocating for in-center hemodialysis patients via anonymous survey
Source: Medicine (Baltimore). 2022 Oct 14;101(41):e30937. doi: 10.1097/MD.0000000000030937 (PMC9575770; doi:10.1097/MD.0000000000030937)
Supplement: Supplementary file 1 [file medi-101-e30937-s001.pdf]

Supplementary data analysis

We evaluated the survey results in a number of ways including simple distributions and counts univariately question by question. As with any user survey, there are individuals who are generally positive on all questions and a few who are thoroughly negative. It is of course difficult to assess whether individuals who are positive or negative to nearly all the questions are just either content or malcontent, or are simply trying to quickly finish a questionnaire. It is well known that satisfaction questionnaires often record a large number of positive responses. To assess how much consistent answering across the board was present in the survey, we created binary variables for each question on the 5-point Likert scale: marking a score of 4 (Good) or 5 (Excellent) to be coded as 1 (highly positive), and a score of anything less than 4 (1: Very poor, 2: Poor, or 3: Neutral) to be coded as 0 (not positive). We then compared the sum of scores overall, and within the 14 satisfaction questions and within the 10 education questions, individually. In addition, we looked for what might be considered behaviorally more truthful responses by comparing pairs of questions looking at discordant responses within the individual satisfaction and education sections. We also felt that the identification of the informative questions would come from the discordant responses between certain questions. We compared and tested these discordant pairs, which we felt might be identifying areas that may need improvement and are more indicative of potential factors on which to intervene, using McNemar's test. P values < 0.05 were considered statistically significant. No adjustments were made for multiple comparisons, as we were looking for consistency rather than simply statistical significance.

Details regarding individual scores where responses differed amongst the satisfaction questions and education questions

In terms of individual scores where responses were discordant in high responses or low responses between the satisfaction questions: 12 individuals who provided a high score (4 or 5) with regards to ‘overall care’ reported a low score (score < 4) to being ‘listened to by staff’, compared to 26 individuals who with regards to ‘overall care’ reported a low score but scored high with being ‘listened to by staff’ (p=0.0336). Similarly, the same pattern was seen for individuals who were satisfied with ‘overall care’ versus being ‘explained by physicians’ in that those 14 individuals reported high scores for ‘overall care’ but low scores for ‘explained by physicians’ compared to 28 who scored low for ‘overall care’ but scored high with being ‘explained by physicians’ (p=0.0436). Amongst those with favorable ‘overall care’, 42 were unfavorable in response to the ‘time spent by the doctor’ question compared to only 24 who scored low on ‘overall care’, but scored high with ‘time spent by the doctor’ (p=0.0356). Similarly, amongst those who reported a high score regarding satisfaction with dialysis ‘improving quality of life’, 48 individuals scored low on ‘time spent by the doctor’ compared to 26 individuals who scored low with dialysis ‘improving quality of life’ but scored high with ‘time spent by the doctor’ (p=0.0141). Similar discordant responses were reported for ‘commute’ and addressal of ‘pain or discomfort’ with 46 individuals scoring high with ‘commute’ but scored low with addressal of ‘pain or discomfort’ and 27 individuals scoring low with ‘commute’ but scoring high with addressal of ‘pain or discomfort’ (p=0.0344). Those scoring high with regards to ‘commute’ showed again more dissatisfaction by scoring low with ‘time spent by the doctor’ (n=41), compared with only 16 individuals who scored low regarding the ‘commute’ question and scoring high with ‘time spent by the doctor’ (p=0.0013). More individuals scored high with ‘cleanness’ of dialysis unit but scored low with ‘time spent by the doctor’ (n=42), compared to 21 individuals who scored low with regards to ‘cleanness’ but scored high with ‘time spent by the doctor’ (p=0.0111). The response to ‘listening’ by doctors was discordant with more individuals scoring high in this parameter but scoring low with regards to addressal of ‘pain or discomfort’ (n=37), compared to those scoring low with ‘listening’ by doctors component but scoring high with addressal of ‘pain or discomfort’ (n=11) (p=0.0002). ‘Listening’ by doctor and ‘time spent by the doctor’ were again discordant for satisfaction (high score) with regards to ‘listening’ by doctor (n=41) but dissatisfied (low score) with ‘time spent by the doctor’ compared to only 9 who were dissatisfied with ‘listening’ by doctor but satisfied with ‘time spent

by the doctor' (p <0.0001). 'Listening' by doctor was rated satisfactory in 29 individuals compared to 15 who were dissatisfied with 'listening' by doctor (p=0.0488). Thirty two individuals reported satisfaction with 'listening' by doctor but expressed dissatisfaction with 'privacy', compared to only 10 individuals who reported dissatisfaction with 'listening' by doctor and satisfaction with 'privacy' (p=0.0009). Thirty eight individuals reported satisfaction with 'explanation' by doctor who were dissatisfied with addressal of 'pain or discomfort', compared to 12 individuals dissatisfied with 'explanation' by doctor but satisfied with addressal of 'pain or discomfort' (p=0.0003). Forty three individuals were satisfied with 'explanation' by doctor, but dissatisfied with 'time spent by the doctor', compared with 11 who were dissatisfied with 'explanation' by doctor and satisfied with 'time spent by the doctor' (p=0.0001). Similarly, 42 individuals satisfied with 'cleanness' were dissatisfied with 'time spent by doctor' compared to 21 vice versa (p=0.0111).

In terms of individual scores where responses were discordant in high responses or low responses between the 10 education based questions, discordant responses were found which seemed to indicate the areas in which education might be desired. With regards to 'enough dialysis' compared to 'education about kidney transplantation', 40 individuals reported a high score with regards to 'enough dialysis' but scored low with regards to education about 'kidney transplantation', compared to only 17 individuals who scored high with regards to education about 'kidney transplantation' but scored low regarding 'enough dialysis' component (p<0.0032). Similarly the discordancy was seen with regards to education pertaining to 'enough dialysis' versus 'home dialysis', 32 versus 16 individuals respectively, (p=0.0293); 'enough dialysis' information versus 'palliative care or hospice' with 76 individuals feeling adequate 'enough dialysis' education was provided (scored high), with only 6 individuals who felt not 'enough dialysis' education was provided (scored low) compared to whether adequate 'palliative care or hospice' information was provided (p<0.0001); comparing education on 'enough dialysis' to education on 'cardiovascular disease affecting dialysis patients', again more (n = 54 vs 13 individuals) felt they were provided 'enough dialysis' education (scored high) but not regarding education (scored low) on 'cardiovascular disease affecting dialysis patients' (p<0.0001). In terms of education with regards to 'elevated phosphorus', more individuals (n=13) felt they were given appropriate educational information (scored high) when compared to education about 'kidney transplantation' (p<0.0001),

‘home dialysis’ (p=0.0031), ‘palliative care or hospice’ (p<0.0001), and ‘cardiovascular disease affecting dialysis patients’ (p<0.0001). Similarly information on ‘high dietary potassium’ was also more commonly provided (high score) than information regarding ‘kidney transplantation’ (p=0.0007), ‘home dialysis’ (p=0.0186), ‘palliative care or hospice’, or ‘cardiovascular disease affecting dialysis patients’ (p<0.0001). When asked if the participant knew about ‘medicines given during dialysis’, again more frequently they scored high over other education based questions: ‘kidney transplantation’ (p=0.0365), ‘palliative care or hospice’ (p<0.0001), and ‘cardiovascular disease affecting dialysis patients’ (p<0.0001). When asked if participants felt that results of their blood tests were explained in a way that they understood, significantly more individuals felt they were educated on this (high score) compared to ‘kidney transplantation’ (p=0.0006), ‘home dialysis’ (p=0.0259), ‘palliative care or hospice’ (p<0.0001), and ‘cardiovascular disease affecting dialysis patients’ (p<0.0001). When comparing ‘kidney transplantation’ education to ‘palliative care or hospice’, more participants felt they were provided appropriate education (scored high) regarding ‘kidney transplantation’ (n = 57) compared to only 10 individuals who felt they were not given sufficient ‘kidney transplantation’ education by scoring low, but offered sufficient ‘palliative care or hospice’ education by scoring high in this component (p<0.0001). Education regarding ‘kidney transplantation’ was also rated higher with regards to information provided when compared to education on ‘cardiovascular disease affecting dialysis patients’ (p=0.0247), and surprisingly ‘dietary counselling’ (p=0.0365). ‘Home dialysis’ education was rated more adequately (scoring high) when compared to education provided regarding ‘palliative care or hospice’ (p<0.0001), or ‘cardiovascular disease affecting dialysis patients’ (p=0.0019). ‘Palliative care or hospice’ education was scored high less often than ‘cardiovascular disease affecting dialysis patients’ with 59 individuals having more positive responses for ‘palliative care or hospice’ compared to only 5 individuals who rated ‘palliative care or hospice’ with a high score but scoring low for ‘home dialysis’ (p<0.0001). ‘Palliative care or hospice’ scored high fewer times when compared to ‘cardiovascular disease affecting dialysis patients’ (p=0.0005), and ‘dietary counselling’ (p<0.0001). Finally, ‘cardiovascular disease affecting dialysis patients’ was also scored high fewer times than ‘dietary counselling’ (p<0.0001).
