# Supplementary material for: Advocating for in-center hemodialysis patients via anonymous survey
Source: Medicine (Baltimore). 2022 Oct 14;101(41):e30937. doi: 10.1097/MD.0000000000030937 (PMC9575770; doi:10.1097/MD.0000000000030937)

Supplemental Figure 1 – Frequency Distribution of Number of Questions being Scored High for Satisfaction and Education Combined. High score entails either 4 (Good) or 5 (Excellent) in questions rated using a 5-point Likert scale (1 = Very poor; 2 = Poor; 3 = Neutral; 4 = Good; 5 = Excellent).

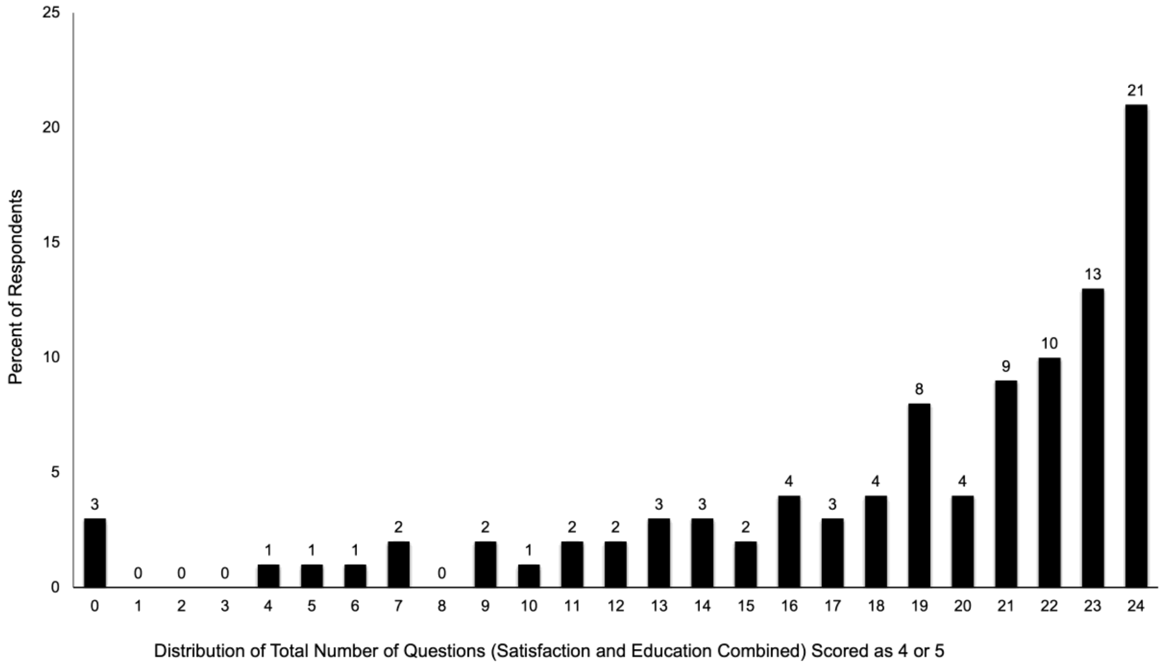

Supplement: Supplementary file 2 [file medi-101-e30937-s002.pdf]
